# Supplementary material for: Predictors of binge drinking among adults in Autonomous Province of Vojvodina, Serbia: Data from the population-based behavioral risk-factors surveillance system
Source: PLoS One. 2026 May 29;21(5):e0350125. doi: 10.1371/journal.pone.0350125 (PMC13221055; doi:10.1371/journal.pone.0350125)
Supplement: Attachment — Submitted filename: Review Comments Binge Drinking Study.docx [file pone.0350125.s001.docx]

# Review Comments: Predictors of Binge Drinking Study

Each comment includes an approximate page and line reference:

1. [Page 1, lines 1–10] The title is clear but could be improved by explicitly noting that the study uses population-based surveillance data, which strengthens its methodological framing.

2. [Page 1, lines 14–22] Ensure consistent use of past tense throughout the abstract to maintain scientific formality and grammatical precision.

3. [Page 1, lines 23–37] The repetition of 'higher likelihoods of binge drinkers' could be condensed into a single, more fluid sentence to enhance readability.

4. [Page 1, line 39] Grammar: add the article 'a' in 'which is a public health priority.'

5. [Page 2, lines 55–60] Clarify the rationale for focusing on Vojvodina, linking to regional health priorities or previous findings to strengthen contextual justification.

6. [Page 2, lines 72–83] The shift from global to Serbian context is abrupt. Consider adding a bridging sentence that connects international evidence to local relevance.

7. [Page 2, lines 87–95] Separate the discussion of individual versus societal factors to improve conceptual clarity.

8. [Page 2, line 111] Use 'Autonomous Province of Vojvodina' consistently instead of 'Autonomy Province' for accuracy.

9. [Page 3, lines 120–126] Move the sentence on study period to the start of the Methods section for logical flow.

10. [Page 3, lines 129–133] Clarify whether sampling included all adults or only healthcare users; this affects representativeness.

11. [Page 3, lines 143–149] The detailed description of healthcare services can be shortened; focus on sampling logic.

12. [Page 3, lines 155–162] Clarify the meaning of 'precision of 1.5%'—cite reference or formula used for calculation.

13. [Page 4, lines 170–180] The ethics paragraph is comprehensive but overly long. Consider condensing without losing essential information.

14. [Page 4, line 185] Replace 'Data were completed' with 'Data were collected' for accuracy.

15. [Page 4, lines 186–190] Add a short note on data security or storage protocols to reinforce data integrity.

16. [Page 5, lines 215–217] Rephrase the BRFSS definition slightly to demonstrate adaptation to local Serbian context.

17. [Page 5, lines 225–228] Clarify whether the 30-day reference period applies uniformly to all variables.

18. [Page 6, lines 235–239] Briefly justify inclusion of explanatory variables—are they theory-driven or empirically based?

19. [Page 6, lines 250–253] Mention statistical software earlier and describe how missing data were handled beyond simple exclusion.

20. [Page 6, line 255] Use 'categorical data' instead of 'attributive data' for proper statistical terminology.

21. [Page 7, lines 270–278] Clarify whether data were weighted to represent the population or analyzed as raw sample values.

22. [Page 8, Table 1] Ensure uniform rounding and consistent presentation of decimals across columns.

23. [Page 9, lines 360–368] Standardize the reporting of confidence intervals to one decimal place (e.g., 29.1–33.7).

24. [Page 10, lines 390–400] Avoid repeating identical odds ratios across genders; consider summarizing concisely.

25. [Page 11, lines 430–440] Include comparison with regional or Eastern European studies to enhance contextual relevance.

26. [Page 12, lines 472–478] Long sentences reduce readability; divide complex arguments into shorter, clearer parts.

27. [Page 13, lines 510–520] Acknowledge potential cross-country differences in educational classifications when comparing results.

28. [Page 14, lines 560–570] Discuss possible bidirectional mechanisms between physical activity and alcohol use for more depth.

29. [Page 15, lines 605–615] Highlight that causality cannot be inferred due to the cross-sectional study design.

30. [Page 16, lines 640–660] Tighten the conclusion by avoiding repetition and clearly restating key implications for practice and research.

## General Summary Comment

Overall, this is a well-conceived and relevant study addressing an important public health issue. The manuscript is methodologically sound and provides valuable insights into binge drinking patterns in Vojvodina. To further strengthen the manuscript, improvements are recommended in language flow, section transitions, and methodological clarity. Enhancing consistency in academic language, tightening redundancies, and expanding contextual discussion will significantly improve the manuscript’s readability and impact.
